# Prognostic implication of stress hyperglycemia in patients with acute coronary syndrome undergoing percutaneous coronary intervention

**DOI:** 10.1186/s12933-023-01790-y

**Published:** 2023-03-21

**Authors:** Man Wang, Wen Su, Ning Cao, Hui Chen, Hongwei Li

**Affiliations:** 1 Department of Cardiology, Cardiovascular Center, Beijing Friendship Hospital, Capital Medical University, No.95, Yongan Road, Xicheng District, Beijing, 100050 People’s Republic of China; 2 Beijing Key Laboratory of Metabolic Disorder Related Cardiovascular Disease, Beijing, China

**Keywords:** Stress hyperglycemia, Glucose/glycated albumin ratio, Acute coronary syndrome, Percutaneous coronary intervention, Prognosis

## Abstract

**Background:**

It is now understood that stress hyperglycemia is associated with adverse outcomes in hospitalized patients. Herein, we aimed to investigate the association between stress hyperglycemia and mortality risk in acute coronary syndrome (ACS) patients who underwent percutaneous coronary intervention (PCI).

**Methods:**

This cohort study comprised 5190 ACS patients who underwent PCI from the Cardiovascular Center Beijing Friendship Hospital Database Bank (CBDBANK) from January 2013 to January 2021. Stress hyperglycemia was defined by the glucose/glycated albumin (GA) ratio, calculated as admission fasting plasma glucose divided by GA. The patients were divided into four groups according to glucose/GA ratio quartiles (Q1-Q4). Cox proportional hazards regression and restricted cubic spline were used to evaluate the association between glucose/GA ratio and all-cause and cardiovascular mortality.

**Results:**

During a median follow-up of 4.0 years, the number of all-cause deaths was 313 (6.0%) and cardiovascular-associated deaths was 177 (3.4%). After adjustment for potential confounders, the risk of all-cause mortality increased in the lowest (HR, 1.43; 95% CI, 1.01–2.03) and highest (HR, 1.51; 95% CI, 1.03–2.21) glucose/GA ratio quartiles compared to Q2. The restricted cubic splines showed that the association between glucose/GA ratio and all-cause mortality was U-shaped after full adjustment (*P*_nonlinear_ = 0.008). Similar results were observed for cardiovascular mortality. In subgroup analyses according to diabetes status, the U-shaped relationship was only significant in patients with diabetes mellitus.

**Conclusion:**

In ACS patients undergoing PCI, low and high glucose/GA ratio values were associated with an increased all-cause and cardiovascular mortality, especially in those with diabetes mellitus.

**Supplementary Information:**

The online version contains supplementary material available at 10.1186/s12933-023-01790-y.

## Introduction

Stress hyperglycemia refers to the transient elevation of blood glucose levels in patients suffering from acute illnesses, such as acute myocardial infarction (AMI), congestive cardiac failure, and cerebrovascular accidents [1–3]. Previous studies have shown that acute stress hyperglycemia was associated with a poor prognosis in patients with acute coronary syndrome (ACS) [4–7]. However, some other studies indicated a significant association between low glycemic levels and adverse outcomes which may provoke confusion [8, 9]. Although patients with previously known diabetes mellitus reportedly have a worse clinical outcome [10–12], it remains controversial how stress hyperglycemia may affect the prognosis of ACS patients with different diabetic status [13, 14].

Different definitions of stress hyperglycemia have been used in the literature based on fasting or random glucose levels [7, 15], which failed to reflect the chronic glycemic levels. Recently, novel markers have been proposed to reflect true acute hyperglycemic status. Most of these markers estimate the average glucose level from glycosylated hemoglobin (HbA1c) [4, 6, 8, 16]. Nevertheless, one recent study used the ratio of fasting plasma glucose (FPG) to glycated albumin (GA) to assess stress hyperglycemia considering the background glucose level before the onset of the acute event [17]. GA is a measure of the mean plasma glucose level over approximately 2–3 weeks, which is shorter than HbA1c, and may reflect glycemic control under conditions with rapid changes in glycemia [18]. Besides, GA is not influenced by conditions such as chronic kidney disease (renal anemia), or hemorrhage which affect erythrocyte lifespan [19]. Thus, GA may provide more accurate information on the actual status of glycemic control compared with HbA1c. However, the association between stress hyperglycemia defined as the ratio of glucose/GA and mortality risk of patients with ACS who underwent percutaneous coronary intervention (PCI) remains unknown, warranting further research.

Therefore, the present study aimed to investigate whether stress hyperglycemia, measured by glucose/GA ratio, could predict mortality in ACS patients with or without diabetes who underwent PCI.

## Methods

### Study population

**Fig. 1:**
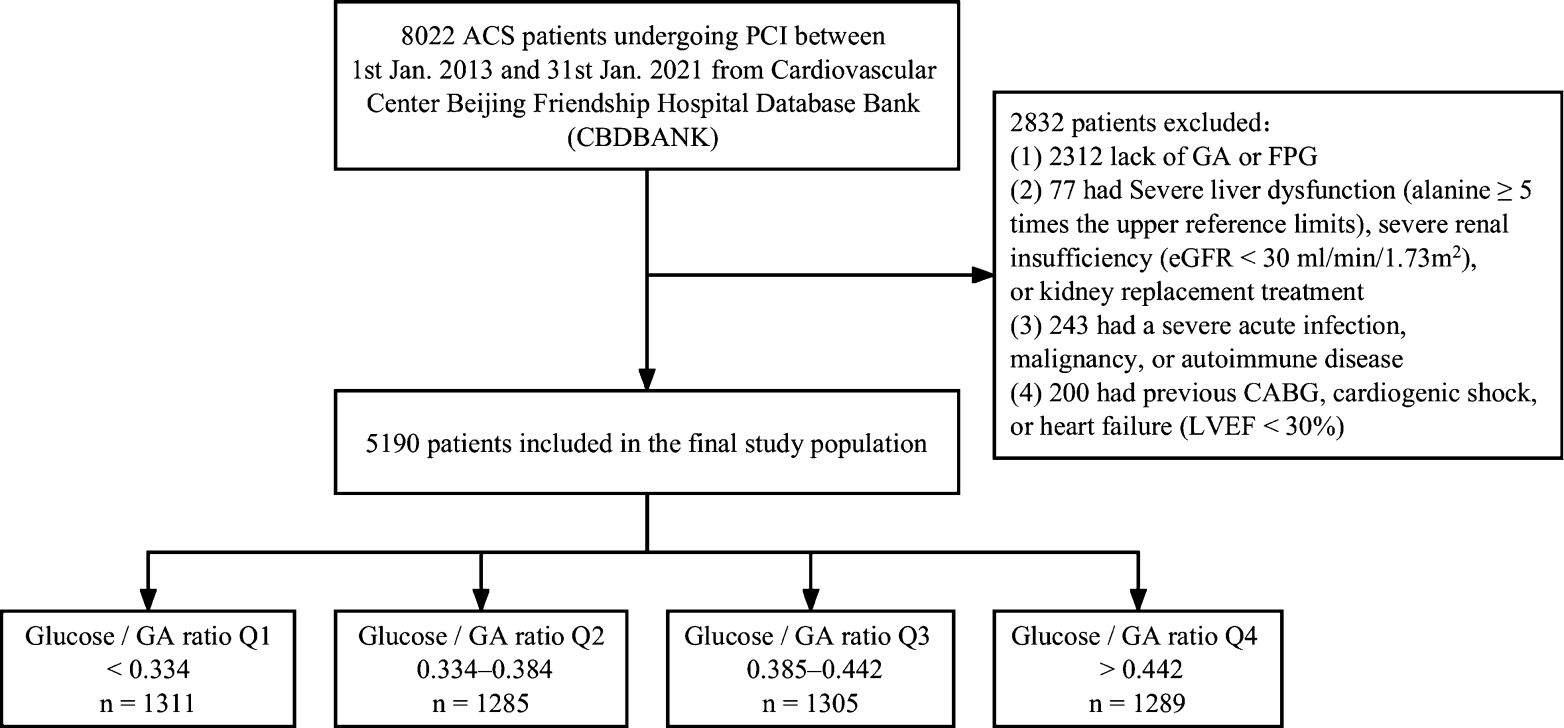
Flowchart for the enrollment of the study population

The Cardiovascular Center Beijing Friendship Hospital Database Bank (CBDBANK) is a large prospective cohort study containing patients diagnosed with ACS from the Department of Cardiology of Beijing Friendship Hospital. Patients with ACS (ST-segment elevation myocardial infarction [STEMI], non-ST-segment elevation myocardial infarction [NSTEMI], and unstable angina [UA]) were diagnosed based on relevant guidelines [20, 21]. A total of 8022 patients were diagnosed with ACS and underwent PCI from January 2013 to January 2021. 2832 patients were excluded according to the following exclusion criteria: (1) lack of GA, FPG, or follow-up data; (2) severe liver dysfunction (alanine ≥ 5 times the upper reference limits), severe renal insufficiency (estimated glomerular filtration rate [eGFR] < 30 ml/min/1.73m^2^), or kidney replacement treatment; (3) severe acute infection, malignancy, or autoimmune disease; (4) previous coronary artery bypass grafting (CABG), cardiogenic shock (defined as systolic blood pressure [SBP] < 90 mmHg for ≥ 30 min or catecholamines to maintain SBP > 90 mmHg, clinical pulmonary congestion and impaired end-organ perfusion [altered mental status, cold/clammy skin and extremities, urine output < 30 ml/h, or lactate > 2.0 mmol/L], or class IV according to the Killip classification), or heart failure (left ventricular ejection fraction [LVEF] < 30%). Finally, 5190 patients were included in this study (Fig. 1). The study was approved by the Ethics Committee of Beijing Friendship Hospital, Capital Medical University, and was conducted in accordance with the Declaration of Helsinki.

### Treatment and procedure

Coronary angiography and PCI were implemented according to relevant guidelines [22]. All patients received a 300 mg loading dose of aspirin, a 300 to 600 mg loading dose of clopidogrel (or 180 mg of ticagrelor), and 70–100 IU/kg unfractionated heparin. PCI was performed using 6 or 7 Fr guiding catheters via the radial artery approach according to the standard techniques by experienced cardiologists. Patients were treated with predilatation and new-generation drug-eluting stents whenever possible. Standard medication after PCI was continued before discharge, including the maintenance dose of aspirin (100 mg/day), clopidogrel (75 mg/day) or ticagrelor (180 mg/day), statin, angiotensin-converting enzyme inhibitors (ACEI) or angiotensin II receptor blockers (ARB), and beta-blockers.

### Assessment of stress hyperglycemia

Overnight fasting venous blood samples were drawn from patients within 24 h after admission and immediately transferred to the central laboratory (Beijing Friendship Hospital) for testing GA and FPG using standard laboratory techniques. Detailed workflow for blood sample collection is shown in Additional file 1: Figure S1. The GA level was presented as a percentage of total serum albumin. Stress hyperglycemia was defined by the glucose/GA ratio [17], which was calculated by using the following equation: glucose/GA ratio = admission FPG (mmol/L)/GA (%). The use of FPG as the numerator instead of admission random blood glucose was based on the fact that it had a greater prognostic value in patients with acute cardiovascular disease [3, 23, 24], was almost unaffected by food or other sugary infusions [3, 25] and exhibited little interindividual heterogeneity [24]. The patients were divided into quartiles of glucose/GA ratio (Q1 < 0.334, Q2 = 0.334–0.384, Q3 = 0.385–0.442, Q4 > 0.442) for further analysis.

### Follow-up and outcome

Relevant information regarding cardiovascular events during hospitalization was confirmed based on their medical records. Clinical follow-up was performed at 1, 6, and 12 months and every year after discharge by telephone interview or outpatient follow-up. The primary endpoint was all-cause mortality during hospitalization and over the follow-up period. Cardiovascular mortality was a secondary outcome. Cardiovascular death was defined as death caused by stroke, AMI, heart failure, or documented sudden cardiac death.

### Covariates

Baseline characteristics, including demographic information (age, sex), medical history, lifestyles (smoking and drinking status [none, ever, current], body mass index [BMI]), laboratory results, and in-hospital therapy were collected from hospital records. The medical history included the presence of comorbidities, including diabetes, hypertension, dyslipidemia, previous coronary heart disease, and chronic kidney diseases. The diagnostic criteria for diabetes include: (1) previously diagnosed diabetes under treatment of antidiabetic medication; (2) the typical symptoms of diabetes with an FPG ≥ 7.0 mmol/L and/or random blood glucose ≥ 11.1 mmol/L and/or 2-h blood glucose after oral glucose tolerance test ≥ 11.1 mmol/L. Hypertension was defined as SBP ≥ 140 mmHg and/or diastolic blood pressure ≥ 90 mmHg three times on different days and/or under antihypertensive treatments.

Overnight fasting blood samples were obtained and tested for total cholesterol (TC), low-density lipoprotein cholesterol (LDL-C), triglyceride, high-density lipoprotein cholesterol (HDL-C), hemoglobin, albumin, high-sensitivity C-reactive protein (hs-CRP), cardiac troponin I (cTnI), N-terminal pro-B-type natriuretic peptide (NT-proBNP), and creatinine in the central laboratory by standard methods. Dyslipidemia was defined as TC > 5.18 mmol/L (200 mg/dL), LDL-C > 3.37 mmol/L (130 mg/dL), triglyceride > 1.72 mmol/L (150 mg/dL), HDL-C < 1.0 mmol/L (40 mg/dL), and/or previous use of lipid-lowering agents. The eGFR was calculated using the Modification of Diet in Renal Disease (MDRD) formula: eGFR (mL/min/1.73 m^2^) = 175 × (Scr)^−1.154^ × (Age)^−0.203^ × (0.742 if female) × (1.212 if African American) [26]. Echocardiograms were performed by cardiologists or certified sonographers, and the LVEF was assessed using the Simpsons method. Medications were obtained directly from the medical records, including aspirin, clopidogrel or ticagrelor, β-blocker, ACEI or ARB, and statins.

### Statistical analysis

Continual variables were presented as means and standard deviation (SD) or median (interquartile ranges [IQR]) and were compared by one-way ANOVA or Kruskal–Wallis H test. Categorical variables were reported as frequency (percentage) and compared by chi-square or Fisher exact test.

Person-years was calculated from baseline to the date of death, loss to follow-up, or the end of follow-up (31 March 2021), whichever came first. Cox proportional hazards regression models were used to calculate adjusted hazard ratios (HRs) and 95% confidence intervals (CIs) for glucose/GA ratio quartiles and mortality. We used three models progressively adjusted for confounders known to influence the prognosis of ACS. Model 1 was adjusted for age and sex. Model 2 included Model 1 variables plus BMI, smoking status, diabetes, hypertension, dyslipidemia, previous myocardial infarction, previous PCI, previous stroke, and AMI. Model 3 included Model 2 variables plus left main coronary artery or three‑vessel disease, eGFR, SBP, heart rate, LVEF < 50%, hs-CRP, albumin, hemoglobin, ACEI/ARB at discharge, and β-blocker at discharge. Adjusted survival curves were performed based on the multivariable Cox regression (Model 3) for describing all-cause and cardiovascular mortality according to the glucose/GA ratio categories [27]. We additionally utilized restricted cubic splines based on Cox models to depict detailed descriptions of the dose–response curves between glucose/GA ratio, all-cause mortality, and cardiovascular mortality [28]. The restricted cubic splines were fitted with 3 knots placed at the 10th, 50th, and 90th percentiles across the range of glucose/GA ratios. Wald tests were used to evaluate the statistical significance (at the 0.05 level) of the overall association and for the nonlinearity of the risk curves. To explore the joint effects of diabetes and glucose/GA ratio in predicting event rates, we determined the incidence rate within each subgroup defined by glucose/GA ratio categories and diabetes status (with or without). Subgroup analyses were conducted to evaluate the association between glucose/GA ratio and mortality according to diabetes status (with or without), age group (< 65 years or ≥ 65 years), sex (male or female), BMI group (< 25 kg/m^2^ or ≥ 25 kg/m^2^), and ACS status (UA or AMI). The *P* values for interactions between categories of glucose/GA ratio and diabetes status, age, sex, BMI, or ACS status for the association of outcomes were also estimated using the Wald χ^2^ test by adding an interaction term (i.e., glucose/GA ratio × diabetes status) in the multivariable models. Finally, we did a sensitivity analysis using E-values to assess how strongly associated unmeasured confounders would need to be with exposure and outcome to potentially fully explain observed non-null association [29].

The data analysis was performed using Stata software, version 17.0 (StataCorp LP, College Station, TX, USA), and R software, version 4.1.2 (R Foundation for Statistical Computing). A two-sided *P*-value < 0.05 was statistically significant.

## Results

A total of 5190 patients were analyzed in the present study with a mean age of 63.4 years, with male predominance (71.6%), and 45.0% presented with AMI. The median glucose/GA ratio was 0.384 (IQR, 0.333–0.442). Table 1 shows patient characteristics stratified by the glucose/GA quartiles. Patients with higher glucose/GA ratio tended to be younger, male and current smokers. Besides, a higher proportion of dyslipidemia and STEMI and higher levels of FPG, hemoglobin, eGFR, hs-CRP, peak cTnI, triglyceride, TC, and LDL-C were observed in this group.

**Table 1:** Baseline characteristics by quartiles of glucose/GA ratio

	Overall n = 5190	Q1* n = 1311	Q2 n = 1285	Q3 n = 1305	Q4 n = 1289	F/χ^2^ value	*P* value
Clinical characteristics							
Age, year	63.4 ± 10.8	66.5 ± 10.3	63.9 ± 10.3	62.5 ± 10.6	60.6 ± 10.9	72.84	< 0.001
Male, n (%)	3714 (71.6)	871 (66.4)	928 (72.2)	949 (72.7)	966 (74.9)	25.28	< 0.001
BMI, kg/m^2^	25.9 ± 3.4	24.9 ± 3.5	25.6 ± 3.2	26.4 ± 3.3	26.5 ± 3.5	62.73	< 0.001
Heart rate, bpm	71.9 ± 12.7	70.0 ± 11.7	70.5 ± 11.5	72.6 ± 12.8	74.7 ± 14.1	38.08	< 0.001
SBP, mmHg	130.5 ± 19.4	131.6 ± 19.4	129.0 ± 18.6	131.2 ± 18.7	129.9 ± 20.8	4.92	0.002
DBP, mmHg	75.5 ± 11.9	74.7 ± 11.3	75.3 ± 11.8	76.4 ± 11.8	75.5 ± 12.6	4.92	0.002
Diabetes, n (%)	2394 (46.1)	612 (46.7)	455 (35.4)	536 (41.1)	791 (61.4)	193.43	< 0.001
Hypertension, n (%)	3497 (67.4)	906 (69.1)	883 (68.7)	883 (67.7)	825 (64.0)	9.56	0.023
Dyslipidemia, n (%)	4424 (85.2)	1064 (81.2)	1090 (84.8)	1129 (86.5)	1141 (88.5)	30.22	< 0.001
Previous MI, n (%)	468 (9.0)	142 (10.8)	108 (8.4)	108 (8.3)	110 (8.5)	7.09	0.069
Previous stroke, n (%)	802 (15.5)	251 (19.1)	187 (14.6)	197 (15.1)	167 (13.0)	20.76	< 0.001
Previous PCI, n (%)	687 (13.2)	198 (15.1)	170 (13.2)	157 (12.0)	162 (12.6)	6.13	0.11
Current smoker, n (%)	2185 (42.1)	410 (31.3)	548 (42.6)	577 (44.2)	650 (50.4)	103.91	< 0.001
LVEF, %	63.0 ± 8.6	63.8 ± 8.7	64.1 ± 7.6	63.3 ± 8.4	60.7 ± 9.1	41.85	< 0.001
ACS status, n (%)						346.94	< 0.001
UA	2855 (55.0)	874 (66.7)	752 (58.5)	760 (58.2)	469 (36.4)		
NSTEMI	1015 (19.6)	247 (18.8)	258 (20.1)	242 (18.5)	268 (20.8)		
STEMI	1320 (25.4)	190 (14.5)	275 (21.4)	303 (23.2)	552 (42.8)		
Laboratory examinations							
FPG, mmol/L	5.6 (4.9, 7.0)	4.9 (4.5, 5.8)	5.3 (4.8, 6.2)	5.7 (5.1, 6.8)	7.2 (5.9, 9.8)	1303.44	< 0.001
GA, %	15.0 (13.1, 18.4)	16.9 (15.0, 20.6)	14.7 (13.4, 17.1)	13.8 (12.4, 16.5)	13.9 (11.9, 18.3)	593.95	< 0.001
Hemoglobin, g/L	136.4 ± 16.7	131.8 ± 16.1	135.8 ± 16.5	137.8 ± 16.1	140.1 ± 16.9	59.47	< 0.001
eGFR, mL/min/1.73m^2^	113.5 ± 29.4	109.5 ± 28.9	112.7 ± 28.6	115.6 ± 28.1	116.3 ± 31.2	14.04	< 0.001
hs-CRP, mg/L	2.3 (0.9, 7.6)	1.7 (0.7, 5.8)	2.0 (0.8, 7.1)	2.4 (0.9, 7.4)	3.4 (1.3, 10.8)	126.72	< 0.001
Peak cTnI, ng/mL	2.3 (0.1, 12.4)	0.5 (0.0, 3.6)	1.2 (0.0, 8.4)	2.8 (0.1, 10.8)	6.9 (0.8, 27.0)	215.03	< 0.001
Peak NT-proBNP, pg/mL	387.5 (112.0, 1454.0)	339.0 (106.0, 1348.0)	323.0 (100.0, 1125.0)	284.0 (95.7, 1179.0)	719.0 (153.0, 2119.0)	87.79	< 0.001
Triglyceride, mmol/L	1.4 (1.1, 2.0)	1.2 (0.9, 1.7)	1.4 (1.1, 1.9)	1.5 (1.1, 2.1)	1.7 (1.2, 2.5)	321.10	< 0.001
Total cholesterol, mmol/L	4.4 ± 1.1	4.2 ± 1.0	4.3 ± 1.0	4.5 ± 1.1	4.7 ± 1.2	47.05	< 0.001
LDL-C, mmol/L	2.6 ± 0.8	2.4 ± 0.7	2.5 ± 0.7	2.6 ± 0.8	2.7 ± 0.8	43.84	< 0.001
HDL-C, mmol/L	1.0 ± 0.3	1.1 ± 0.2	1.0 ± 0.3	1.0 ± 0.2	1.0 ± 0.3	0.82	0.48
In-hospital treatment, n (%)							
Aspirin	5055 (97.4)	1271 (96.9)	1255 (97.7)	1278 (97.9)	1251 (97.1)	3.48	0.32
Clopidogrel/Ticagrelor	4840 (93.3)	1211 (92.4)	1202 (93.5)	1223 (93.7)	1204 (93.4)	2.28	0.52
β-Blocker	3693 (71.2)	900 (68.6)	874 (68.0)	939 (72.0)	980 (76.0)	25.5	< 0.001
ACEI/ARB	3013 (58.1)	745 (56.8)	725 (56.4)	753 (57.7)	790 (61.3)	7.82	0.050
Statins	4761 (91.7)	1216 (92.8)	1182 (92.0)	1205 (92.3)	1158 (89.8)	8.65	0.034
Angiographic data, n (%)							
LM lesion	545 (10.5)	164 (12.5)	136 (10.6)	131 (10.0)	114 (8.8)	9.70	0.021
Multi-vessel lesion	4204 (81.0)	1040 (79.3)	1034 (80.5)	1062 (81.4)	1068 (82.9)	5.62	0.13
Chronic total occlusion lesion	1791 (34.5)	354 (27.0)	405 (31.5)	424 (32.5)	608 (47.2)	131.53	< 0.001
Target vessel territory, n (%)							
LM	172 (3.3)	54 (4.1)	48 (3.7)	44 (3.4)	26 (2.0)	10.14	0.017
LAD	2569 (49.5)	653 (49.8)	626 (48.7)	646 (49.5)	644 (50.0)	0.48	0.92
LCX	999 (19.2)	256 (19.5)	251 (19.5)	260 (19.9)	232 (18.0)	1.81	0.61
RCA	1576 (30.4)	386 (29.4)	396 (30.8)	391 (30.0)	403 (31.3)	1.24	0.74
Hypoglycemic agents, n (%)							
Metformin	705 (13.6)	173 (13.2)	133 (10.4)	181 (13.9)	218 (16.9)	23.87	< 0.001
Alpha-glucosidase inhibitor	1202 (23.2)	277 (21.1)	230 (17.9)	286 (21.9)	409 (31.7)	77.36	< 0.001
Sulfonylurea	420 (8.1)	99 (7.6)	81 (6.3)	96 (7.4)	144 (11.2)	23.43	< 0.001
DPP-4i	33 (0.6)	9 (0.7)	8 (0.6)	6 (0.5)	10 (0.8)	1.10	0.78
Insulin	510 (9.8)	163 (12.4)	88 (6.8)	93 (7.1)	166 (12.9)	47.20	< 0.001

Values are mean ± SD, n (%), or median (interquartile range)

ACEI, angiotensin-converting enzyme inhibitor; ARB, angiotensin receptor blocker; BMI, body mass index; cTnI, cardiac troponin I; DBP, diastolic blood pressure; DPP-4i, dipeptidyl peptidase-4 inhibitors; eGFR, estimated glomerular filtration rate; FPG, fasting plasma glucose; GA, glycated albumin; HDL-C, high-density lipoprotein cholesterol; hs-CRP, high sensitivity C-reactive protein; LAD, left anterior descending artery; LCX, left circumflex artery; LDL-C, low-density lipoprotein cholesterol; LM, left main coronary artery; LVEF, left ventricular ejection fraction; MI, myocardial infarction; NSTEMI, non-ST-segment elevation myocardial infarction; NT-proBNP, N-terminal pro-B-type natriuretic peptide; PCI, percutaneous coronary intervention; RCA, right coronary artery; SBP, systolic blood pressure; STEMI, ST-segment elevation myocardial infarction; UA, unstable angina

^*^Quartiles of glucose/GA ratio, Q1 < 0.334, Q2 = 0.334–0.384, Q3 = 0.385–0.442, Q4 > 0.442

**Fig. 2:**
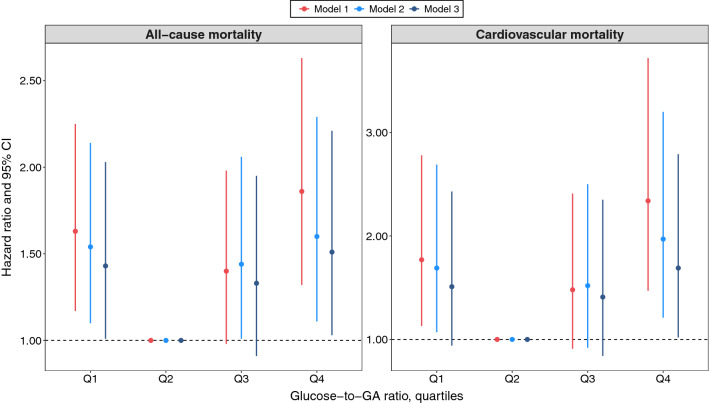
Adjusted hazard ratios for all-cause mortality and cardiovascular mortality according to glucose/GA ratio categories. Model 1 included age and sex. Model 2 included Model 1 variables plus BMI, smoking status, diabetes, hypertension, dyslipidemia, previous myocardial infarction, previous PCI, previous stroke, and AMI. Model 3 included Model 2 variables plus left main coronary artery or three‑vessel disease, eGFR, SBP, heart rate, LVEF < 50%, hs-CRP, albumin, hemoglobin, ACEI/ARB at discharge, and β-blocker at discharge

**Fig. 3:**
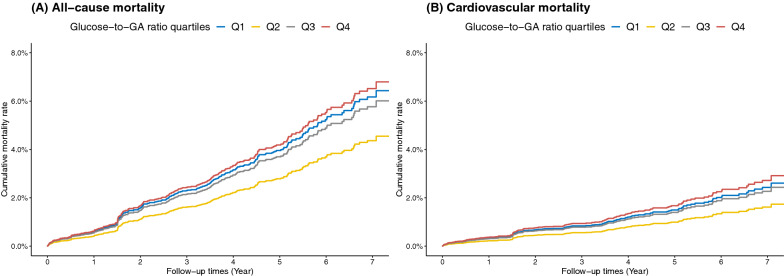
Adjusted Kaplan–Meier curves for all-cause mortality (**A**) and cardiovascular mortality (**B**) according to the glucose/GA ratio categories

During a median follow-up of 4.0 years (IQR 1.1–5.1 years), the number of all-cause deaths and cardiovascular-associated deaths was 313 (6.0%) and 177 (3.4%), respectively. Associations between glucose/GA ratio quartiles and all-cause and cardiovascular mortality are shown in Table 2 and Fig. 2. After adjustment for potential confounders, the risk of all-cause mortality increased in the lowest and highest glucose/GA ratio quartiles, exhibiting a U-shaped relationship between glucose/GA ratio and all-cause mortality. For patients with glucose/GA ratio level < 0.334 (Q1), the fully adjusted HR for all-cause mortality was 1.43 (95% CI, 1.01–2.03) compared to Q2. For patients with glucose/GA ratio level > 0.442 (Q4), the adjusted HR for all-cause mortality was 1.51 (95% CI, 1.03–2.21). Similarly, the incidence rate for cardiovascular mortality increased in patients with the lowest and highest glucose/GA ratio quartiles. After adjusting for covariates in model 3, only the highest glucose/GA ratio level was associated with increased cardiovascular mortality (adjusted HR, 1.69; 95% CI, 1.02–2.79) (Table 2 and Fig. 2). Covariates-adjusted survival curves of time until all-cause and cardiovascular death are shown in Fig. 3. Patients in the lowest and highest glucose/GA ratio groups were associated with higher mortality during follow-up.

**Table 2:** Association between glucose/GA ratio and all-cause and cardiovascular mortality

Outcome	Cases, No	Incidence Rate, per 1000 Person-Years	HR (95% CI)		
			Model 1 ^a^	Model 2 ^b^	Model 3 ^c^
All-cause mortality					
Glucose/GA ratio, quartiles^d^					
Q1	107	24.1	1.63 (1.17–2.25)	1.54 (1.10–2.14)	1.43 (1.01–2.03)
Q2	55	12.2	1 [Reference]	1 [Reference]	1 [Reference]
Q3	71	15.2	1.40 (0.98–1.98)	1.44 (1.01–2.06)	1.33 (0.91–1.95)
Q4	80	18.0	1.86 (1.32–2.63)	1.60 (1.11–2.29)	1.51 (1.03–2.21)
Cardiovascular mortality					
Glucose/GA ratio, quartiles					
Q1	60	13.5	1.77 (1.13–2.78)	1.69 (1.07–2.69)	1.51 (0.94–2.43)
Q2	28	6.2	1 [Reference]	1 [Reference]	1 [Reference]
Q3	38	8.1	1.48 (0.91–2.41)	1.52 (0.92–2.50)	1.41 (0.84–2.35)
Q4	51	11.5	2.34 (1.47–3.72)	1.97 (1.21–3.20)	1.69 (1.02–2.79)

CI, confidence interval; GA, glycated albumin; and HR, hazard ratio

^a^ Model 1 was adjusted for age and sex

^b^ Model 2 was adjusted as model 1 plus BMI, smoking status, diabetes, hypertension, dyslipidemia, previous myocardial infarction, previous PCI, previous stroke, and AMI

^c^ Model 3 was adjusted as model 2 plus left main coronary artery or three‑vessel disease, eGFR, SBP, heart rate, LVEF < 50%, hs-CRP, albumin, hemoglobin, ACEI/ARB at discharge, and β-blocker at discharge

^d^ Quartiles of glucose/GA ratio, Q1 < 0.334, Q2 = 0.334–0.384, Q3 = 0.385–0.442, Q4 > 0.442

**Fig. 4:**
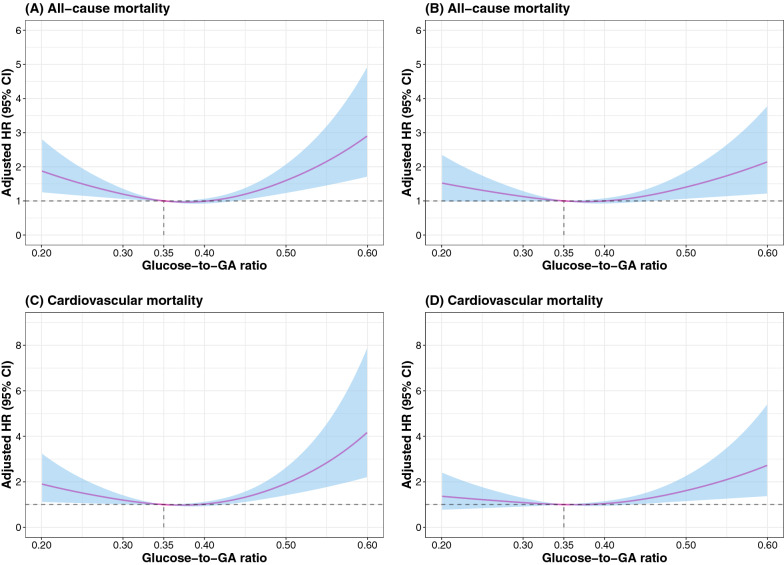
Restricted cubic spline analysis for association between the glucose/GA ratio and all-cause mortality or cardiovascular mortality with the adjustment of covariates in Model 1 (**A** and **C**) or Model 3 (**B** and **D**). Model 1 included age and sex. Model 3 included Model 1 variables plus BMI, smoking status, diabetes, hypertension, dyslipidemia, previous myocardial infarction, previous PCI, previous stroke, AMI, left main coronary artery or three‑vessel disease, eGFR, SBP, heart rate, LVEF < 50%, hs-CRP, albumin, hemoglobin, ACEI/ARB at discharge, and β-blocker at discharge

The dose–response relationships between glucose/GA ratio level and all-cause and cardiovascular mortality using restricted cubic splines are shown in Fig. 4. The association between glucose/GA ratio and all-cause mortality was U-shaped after adjusting for variables in Model 3 (*P*_nonlinear_ = 0.008). Similar results were observed for the association between glucose/GA ratio and cardiovascular mortality (*P*_nonlinear_ = 0.028).

**Fig. 5:**
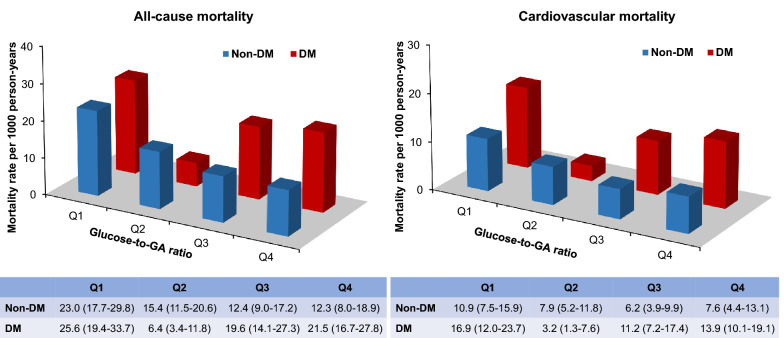
Incidence rates per 1000 person-years of all-cause mortality and cardiovascular mortality according to the combination of glucose/GA ratio quartiles and diabetes status

We subsequently explored the effect modification of diabetes status on the association between glucose/GA ratio and mortality in subgroup analyses. The joint association of diabetes status and glucose/GA ratio quartiles with all-cause and cardiovascular mortality is depicted in Fig. 5. In patients with diabetes, the all-cause mortality rate was highest in the Q1 group (25.6; 95% CI, 19.4–33.7/1000 person-years), and followed by the Q4 group (21.5; 95% CI, 16.7–27.8/1000 person-years). In patients without diabetes, the all-cause mortality rate was higher in Q1 (23.0; 95% CI, 17.7–29.8/1000 person-years) and Q2 (15.4; 95% CI, 11.5–20.6/1000 person-years) groups. Similar trends were observed when considering cardiovascular mortality (Fig. 5). In the multivariate Cox analysis, higher mortality risk was observed in the lowest and highest glucose/GA ratio groups for patients with diabetes (Table 3). Compared with patients in the Q2 group, the multivariable-adjusted HR for all-cause mortality was 3.19 (95% CI, 1.55–6.56), 3.04 (95% CI, 1.44–6.42) and 3.36 (95% CI, 1.64–6.91) for patients in the Q1, Q3, and Q4 groups. Similarly, the corresponding multivariable-adjusted HR (95% CI) for cardiovascular mortality was 4.18 (1.61–10.88), 3.46 (1.28–9.36), and 3.91 (1.50–10.17) for patients in the Q1, Q3, and Q4 group, respectively. In contrast, this association was non-significant for patients without diabetes (Table 3). Moreover, there was a significant interaction between glucose/GA ratio and diabetes status for all-cause mortality (*P* for interaction = 0.038), but not for cardiovascular mortality (*P* for interaction = 0.061).

**Table 3:** Association between glucose/GA ratio and all-cause and cardiovascular mortality according to diabetes status

Outcome	Diabetes (n = 2394)					No diabetes (n = 2796)				
	Cases, No	Incidence Rate^*^	HR (95% CI)			Cases, No	Incidence Rate	HR (95% CI)		
			Model 1 ^a^	Model 2 ^b^	Model 3 ^c^			Model 1 ^a^	Model 2 ^b^	Model 3 ^c^
All-cause mortality										
Glucose/GA ratio, quartiles^d^										
Q1	50	25.6	3.49 (1.77–6.89)	3.30 (1.67–6.52)	3.19 (1.55–6.56)	57	23.0	1.18 (0.79–1.74)	1.11 (0.74–1.68)	1.10 (0.71–1.70)
Q2	10	6.4	1 [Reference]	1 [Reference]	1 [Reference]	45	15.4	1 [Reference]	1 [Reference]	1 [Reference]
Q3	35	19.6	3.22 (1.59–6.50)	3.14 (1.55–6.38)	3.04 (1.44–6.42)	36	12.4	0.96 (0.62–1.49)	1.04 (0.67–1.62)	0.98 (0.61–1.58)
Q4	59	21.5	3.82 (1.95–7.47)	3.37 (1.70–6.65)	3.36 (1.64–6.91)	21	12.3	1.24 (0.73–2.09)	1.14 (0.67–1.94)	1.11 (0.64–1.94)
Cardiovascular mortality										
Glucose/GA ratio, quartiles										
Q1	33	16.9	4.58 (1.79–11.75)	4.40 (1.71–11.30)	4.18 (1.61–10.88)	27	10.9	1.08 (0.61–1.88)	1.02 (0.57–1.83)	0.87 (0.46–1.61)
Q2	5	3.2	1 [Reference]	1 [Reference]	1 [Reference]	23	7.9	1 [Reference]	1 [Reference]	1 [Reference]
Q3	20	11.2	3.68 (1.38–9.80)	3.51 (1.31–9.43)	3.46 (1.28–9.36)	18	6.2	0.95 (0.51–1.76)	1.09 (0.58–2.04)	1.01 (0.52–1.97)
Q4	38	13.9	4.96 (1.95–12.62)	4.30 (1.67–11.04)	3.91 (1.50–10.17)	13	7.6	1.52 (0.77–3.03)	1.51 (0.74–3.07)	1.32 (0.63–2.75)

CI, confidence interval; GA, glycated albumin; and HR, hazard ratio

^*^per 1000 person-years

^a^Model 1 was adjusted for age and sex

^b^Model 2 was adjusted as model 1 plus BMI, smoking status, hypertension, dyslipidemia, previous myocardial infarction, previous PCI, previous stroke, and AMI

^c^Model 3 was adjusted as model 2 plus left main coronary artery or three‑vessel disease, eGFR, SBP, heart rate, LVEF < 50%, hs-CRP, albumin, hemoglobin, ACEI/ARB at discharge, and β-blocker at discharge

^d^Quartiles of glucose/GA ratio, Q1 < 0.334, Q2 = 0.334–0.384, Q3 = 0.385–0.442, Q4 > 0.442

*P* for interaction between glucose/GA ratio and diabetes group for all-cause mortality was 0.023 in Model 1, 0.033 in Model 2, and 0.038 in Model 3

*P* for interaction between glucose/GA ratio and diabetes group for cardiovascular mortality was 0.075 in Model 1, 0.088 in Model 2, and 0.061 in Model 3

Subgroup analyses were performed for all-cause mortality by the following variables: age, sex, BMI, and ACS status (Additional file 1: Figure S2–S5). The U-shaped relationship between glucose/GA ratio and all-cause mortality was more pronounced among patients aged < 65 years, females, and patients with BMI < 25 kg/m^2^. However, there was no significant interaction between the glucose/GA ratio and each subgroup for all-cause mortality (all *P* for interaction > 0.05).

In sensitivity analysis, the estimated E-value for the association between glucose/GA ratio and mortality based on the fully adjusted model is shown in Additional file 1: Figures S6 and S7. For all-cause mortality, the E-values were 2.21 and 2.39 for glucose/GA ratio level < 0.334 (Q1) and > 0.442 (Q4) compared to Q2 (Additional file 1: Figure S6). Similarly, the E-values for cardiovascular mortality were 2.39 and 2.77 accordingly (Additional file 1: Figure S7).

## Discussion

In this large, prospective cohort study of patients with ACS undergoing PCI, we provided preliminary evidence that low and high glucose/GA ratio levels were associated with a higher mortality risk, exhibiting a U-shaped relationship. Interestingly, this relationship varied by diabetes status, with a significant association between glucose/GA ratio and mortality in patients with diabetes compared with those without diabetes.

### Stress hyperglycemia in patients with ACS

Stress hyperglycemia has been documented as a strong predictor of adverse outcomes in patients with ACS. It has been reported that stress hyperglycemia is significantly associated with major adverse cardiovascular and cerebrovascular events, irrespective of the diabetes status in STEMI patients undergoing PCI [5]. In addition, stress hyperglycemia ratio (SHR), representing relative hyperglycemia using the ratio of the admission blood glucose to estimated chronic blood glucose, is significantly related to in-hospital mortality in patients with coronary artery disease, especially for those with prediabetes and diabetes [30]. Moreover, it has been reported that, unlike the admission blood glucose, the SHR is an independent predictor of in-hospital mortality after AMI and improves the predictability of prognostic models containing the Global Registry of Acute Coronary Events (GRACE) score [6]. Similarly, Luo et al. reported that adding SHR to the GRACE score significantly improved its post-MI risk stratification performance among patients with diabetes [31]. These studies suggested a strong prognostic value of stress hyperglycemia, which may help identify ACS patients with a higher risk of subsequent adverse outcomes. The present study used a novel index of relative hyperglycemia, the glucose/GA ratio, and evaluated the association between glucose/GA ratio and mortality in ACS patients that underwent PCI.

### Dose–response relationship between hyperglycemia and outcomes

Overwhelming evidence substantiates that a higher level of stress hyperglycemia is significantly associated with adverse outcomes [4–7]. However, most of these studies were conducted without exploring the potential differences among the lower groups of relative hyperglycemia index and ignored the nonlinear relationship. The present study assessed a novel index of relative hyperglycemia, indicating a U-shaped relationship with low and high glucose/GA ratio levels associated with a higher mortality risk, especially in diabetes patients. Another study of 5562 patients with ACS who underwent PCI reported the U-shaped or J-shaped association between SHR and early and late poor prognosis using restricted cubic splines analyses [8]. Notably, the U-shaped or J-shaped association was not significant in the subgroup of patients without diabetes, consistent with our findings [8]. Moreover, Zhou et al. found a U-shaped association between SHR and in-hospital cardiac, kidney, and infectious adverse events in non-surgical hospitalized patients with type 2 diabetes and heart failure [32]. In addition, one study consisting of 3750 AMI patients admitted to 35 hospitals in Japan found that severe hyperglycemia (glucose ≥ 11 mmol/L) and euglycemia (glucose < 7 mmol/L) was associated with higher mortality compared to moderate hyperglycemia (glucose 9 to 11 mmol/L) in patients with a history of diabetes. On the contrary, this relationship was linear in non-diabetic patients, with glucose levels < 6 mmol/L associated with the lowest mortality [9].

It has long been thought that stress hyperglycemia, as an index of disease severity, enables quantification of the degree of acute illness and has a prognostic value. A positive relationship between hyperglycemia and infarct size, reduced LVEF, the severity of microvascular obstruction, and the use of an intra-aortic balloon pump were reported in the AMI population [33–35]. In addition, Goyal et al. documented that a more substantial drop in glucose in the first 24 h after AMI was associated with decreased mortality, which has potential implications for a cause-and-effect relationship between hyperglycemia and increased mortality [36]. However, the U-shaped phenomenon may provoke confusion and challenge the blood glucose management strategy.

### Mechanisms and implications

The mechanisms underlying the U-shaped association between stress hyperglycemia and outcomes in ACS patients remain unknown. It has been reported that mild-to-moderate stress hyperglycemia might play a protective role during the acute phase, especially for ischemia. Following reduced blood flow during ischemia, moderate hyperglycemia (blood glucose of 140 to 220 mg/dL) results in a new glucose balance with a higher blood ‘glucose diffusion gradient’ conducive to maximum cellular glucose uptake [37, 38]. In addition, hyperglycemia may reduce infarct size and improve systolic function by increasing cell survival factors (hypoxia-inducible factor-1α, vascular endothelial growth factor) and decreasing apoptosis [39]. Besides, the diabetic status might modulate this relationship in clinically important ways. Consistent with previous studies [8, 9], the U-shaped relationship between the hyperglycemia index (as glucose/GA ratio in the present study) and mortality was more significant in patients with diabetes. A study involving 44,964 patients admitted to intensive care units suggested that patients with diabetes may benefit from higher glucose target ranges than those without [40]. However, it should be borne in mind that severe stress hyperglycemia may still be harmful. In our study, we found that the inflection points of the glucose/GA ratio for the whole cohort was approximately 0.35, and higher values indicated an elevated risk of mortality. Since the blood glucose level is usually higher in patients with diabetes before ACS, the threshold glucose level associated with deleterious effects might be raised. Indeed, more prospective cohort studies are needed to determine the threshold of stress hyperglycemia, and a more stratified glycemic target should be applied according to the glucose/GA ratio value.

### Strengths and limitations

The strengths of this study included a large sample size, a long follow-up period, and the glucose/GA ratio measures to assess relative acute rises in plasma glucose level compared with premorbid glucose status. To our knowledge, this is the first study to confirm the prognostic value of the glucose/GA ratio in ACS patients who underwent PCI. However, several limitations need to be addressed. First, this was a single-center cohort study in the Chinese population, which limits the generalization of our findings. Besides, given the observational nature of the cohort studies, only the association between the glucose/GA ratio and the outcome was determined rather than a causal relationship. The presence of residual or unmeasured confounding factors, such as the symptom onset to balloon time, could not be entirely ruled out. In addition, FPG and GA levels were only measured once after admission, which could cause potential bias. Additional prospective and mechanistic cohort studies are required to further validate our findings.

## Conclusion

Both low and high glucose/GA ratio values were associated with an increased all-cause and cardiovascular mortality, exhibiting a U-shaped relationship. Indeed, further studies are needed to validate these findings and explore the underlying mechanisms.

## Supplementary Information

**Additional file 1.** Additional figures.

## Data Availability

The datasets are available from the corresponding author on reasonable request.

## Abbreviations

ACS: Acute coronary syndrome
PCI: Percutaneous coronary intervention
CBDBANK: Cardiovascular Center Beijing Friendship Hospital Database Bank
GA: Glycated albumin
AMI: Acute myocardial infarction
HbA1c: Glycosylated hemoglobin
FPG: Fasting plasma glucose
STEMI: ST-segment elevation myocardial infarction
NSTEMI: Non-ST-segment elevation myocardial infarction
UA: Unstable angina
eGFR: Estimated glomerular filtration rate
CABG: Coronary artery bypass grafting
SBP: Systolic blood pressure
LVEF: Left ventricular ejection fraction
ACEI: Angiotensin-converting enzyme inhibitor
ARB: Angiotensin receptor blocker
BMI: Body mass index
TC: Total cholesterol
LDL-C: Low-density lipoprotein cholesterol
HDL-C: High-density lipoprotein cholesterol
hs-CRP: High-sensitivity C-reactive protein
cTnI: Cardiac troponin I
NT-proBNP: N-terminal pro-B-type natriuretic peptide
SD: Standard deviation
HR: Hazard ratio
CI: Confidence interval
IQR: Interquartile range
