# Supplementary material for: Prognostic implication of stress hyperglycemia in patients with acute coronary syndrome undergoing percutaneous coronary intervention
Source: Cardiovasc Diabetol. 2023 Mar 21;22:63. doi: 10.1186/s12933-023-01790-y (PMC10031999; doi:10.1186/s12933-023-01790-y)
Supplement: Supplementary file 1 — Additional file 1. Additional figures. [file 12933_2023_1790_MOESM1_ESM.docx]

**Supplemental material**

**Figure S1. Work-flow chart.**
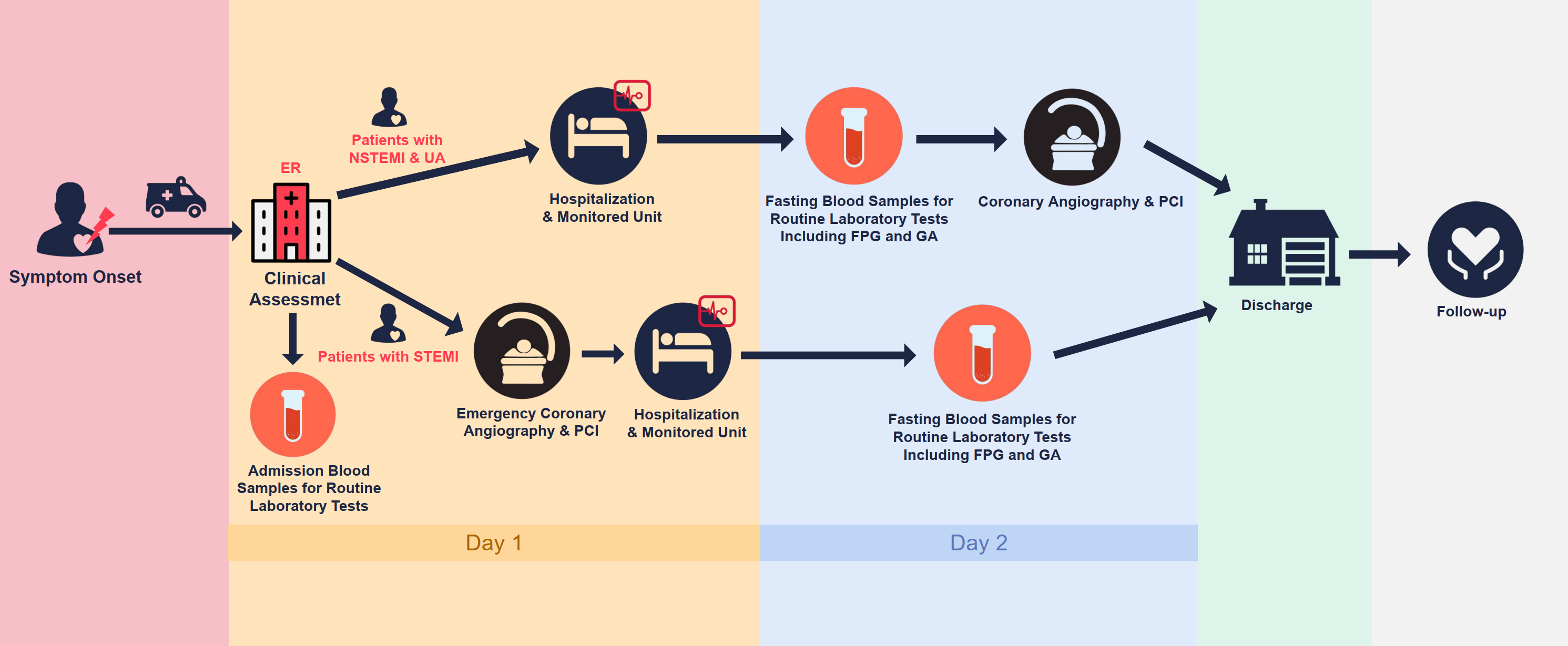


Abbreviations: ER, emergency room; FPG, fasting plasma glucose; GA, glycated albumin; NSTEMI, non-ST-segment elevation myocardial infarction; PCI, percutaneous coronary intervention; STEMI, ST-segment elevation myocardial infarction; UA, unstable angina.

**Figure S2. Subgroup analysis for the association between glucose/GA ratio and all-cause mortality by age groups (< 65 years, ≥ 65 years)**

Notes:

Model 1 was adjusted for gender.

Model 2 was adjusted as model 1 plus body mass index, smoking status, diabetes, hypertension, dyslipidemia, previous MI, previous PCI, previous stroke, and AMI.

Model 3 was adjusted as model 2 plus left main coronary artery or three‑vessel disease, eGFR, systolic blood pressure, heart rate, LVEF < 50%, hs-CRP, albumin, hemoglobin, ACEI/ARB at discharge, and β-blocker at discharge.

*P* for interaction between glucose/GA ratio and age group for all-cause mortality was 0.542 in Model 1, 0.457 in Model 2, and 0.459 in Model 3.

**Figure S3. Subgroup analysis for the association between glucose/GA ratio and all-cause mortality by sex**

Notes:

Model 1 was adjusted for age

Model 2 was adjusted as model 1 plus body mass index, smoking status, diabetes, hypertension, dyslipidemia, previous MI, previous PCI, previous stroke, and AMI.

Model 3 was adjusted as model 2 plus left main coronary artery or three‑vessel disease, eGFR, systolic blood pressure, heart rate, LVEF < 50%, hs-CRP, albumin, hemoglobin, ACEI/ARB at discharge, and β-blocker at discharge.

*P* for interaction between glucose/GA ratio and gender for all-cause mortality was 0.402 in Model 1, 0.263 in Model 2, and 0.195 in Model 3.

**Figure S4. Subgroup analysis for the association between glucose/GA ratio and all-cause mortality by body mass index (< 25 kg/m^2^, ≥ 25 kg/m^2^)**

Notes:

Model 1 was adjusted for age and gender.

Model 2 was adjusted as model 1 plus smoking status, diabetes, hypertension, dyslipidemia, previous MI, previous PCI, previous stroke, and AMI.

Model 3 was adjusted as model 2 plus left main coronary artery or three‑vessel disease, eGFR, systolic blood pressure, heart rate, LVEF < 50%, hs-CRP, albumin, hemoglobin, ACEI/ARB at discharge, and β-blocker at discharge.

*P* for interaction between glucose/GA ratio and body mass index group for all-cause mortality was 0.175 in Model 1, 0.144 in Model 2, and 0.110 in Model 3.

**Figure S5. Subgroup analysis for the association between glucose/GA ratio and all-cause mortality by ACS status**

Notes:

Model 1 was adjusted for age and gender.

Model 2 was adjusted as model 1 plus smoking status, diabetes, hypertension, dyslipidemia, previous MI, previous PCI, and previous stroke.

Model 3 was adjusted as model 2 plus left main coronary artery or three‑vessel disease, eGFR, systolic blood pressure, heart rate, LVEF < 50%, hs-CRP, albumin, hemoglobin, ACEI/ARB at discharge, and β-blocker at discharge.

*P* for interaction between glucose/GA ratio and ACS status for all-cause mortality was 0.916 in Model 1, 0.976 in Model 2, and 0.901 in Model 3.

**Figure S6. Minimum associations with unmeasured confounders required to potentially explain the observed confounder-adjusted association between glucose/GA ratio quartiles and all-cause mortality**

**Figure S7. Minimum associations with unmeasured confounders required to potentially explain the observed confounder-adjusted association between glucose/GA ratio quartiles and** **cardiovascular mortality**
